# Differential ability of three bee species to move genes via pollen

**DOI:** 10.1371/journal.pone.0271780

**Published:** 2023-04-13

**Authors:** Fabiana P. Fragoso, Johanne Brunet

**Affiliations:** 1 Agricultural Research Service Research Participation Program, Oak Ridge Institute for Science and Education (ORISE), Madison, Wisconsin, United States of America; 2 Vegetable Crops Research Unit, United States Department of Agriculture, Agricultural Research Service, Madison, Wisconsin, United States of America; University of Leipzig Faculty of Life Sciences: Universitat Leipzig Fakultat fur Lebenswissenschaften, GERMANY

## Abstract

Since the release of genetically engineered (GE) crops, there has been increased concern about the introduction of GE genes into non-GE fields of a crop and their spread to feral or wild cross-compatible relatives. More recently, attention has been given to the differential impact of distinct pollinators on gene flow, with the goal of developing isolation distances associated with specific managed pollinators. To examine the differential impact of bee species on gene movement, we quantified the relationship between the probability of getting a GE seed in a pod, and the order in which a flower was visited, or the cumulative distance traveled by a bee in a foraging bout. We refer to these relationships as ‘seed curves’ and compare these seeds curves among three bee species. The experiments used *Medicago sativa* L. plants carrying three copies of the glyphosate resistance (GR) allele as pollen donors (*M*. *sativa* is a tetraploid), such that each pollen grain carried the GR allele, and conventional plants as pollen recipients. Different foraging metrics, including the number of GR seeds produced over a foraging bout, were also quantified and contrasted among bee species. The lowest number of GR seeds set per foraging bout, and the GR seeds set at the shortest distances, were produced following leafcutting bee visits. In contrast, GR seeds were found at the longest distances following bumble bee visits. Values for honey bees were intermediate. The ranking of bee species based on seed curves correlated well with field-based gene flow estimates. Thus, differential seed curves of bee species, which describe patterns of seed production within foraging bouts, translated into distinct abilities of bee species to move genes at a landscape level. Bee behavior at a local scale (foraging bout) helps predict gene flow and the spread of GE genes at the landscape scale.

## Introduction

In flowering plants, gene flow occurs predominantly through the dispersal of pollen, and subsequent seed set from pollinated ovules [1, 2]. Gene flow can also occur through the dispersal of seeds [3, 4]. Insects move pollen between flowers, and most flowering plants, including a large number of fruit crops, most vegetable crops when one considers seed production, together with some forage and oil producing crops, are insect-pollinated [5, 6]. As the agents responsible for the transfer of pollen from flower to flower in the majority of angiosperms, pollinators impact the movement of genes via pollen [6–8] and affect plant reproductive success [9, 10].

In agriculture, gene flow can occur among fields of a crop or between a crop and a cross-compatible feral or wild relative [11, 12]. The seed industry has long been interested in limiting gene flow among fields of a crop in order to maintain the genetic purity of crop varieties. Moreover, since the release of genetically engineered (GE) crops, there has been increased concern about the introduction of GE genes into non-GE fields of a crop, and their spread to feral or wild cross-compatible relatives [13–16]. The movement of pollen from a GE to a non-GE variety of the same crop can contaminate seed lots, create adventitious presence (unwanted GE genes in seed lots), and offset coexistence of different markets with serious negative economic impacts [17, 18]. In fact, the organic and export markets have a very low tolerance threshold for GE genes and their presence can disqualify a seed lot for the intended market. In addition, in cross-compatible feral or wild relatives of a crop, the GE gene transmitted from the GE crop via pollen or seeds could increase in frequency with potentially negative consequences [12, 15, 19, 20]. For example, a gene that increases the competitive ability of the plant could increase the weediness and invasiveness of the wild or feral species and consequently reduce the biodiversity of the community [12, 15]. The rapid development of genome editing technologies is expected to increase both the numbers and acreages planted to GE crops, with growing concern for gene flow to feral and wild populations, and for adventitious presence that can disrupt the coexistence of different seed markets (organic, conventional, export) and negatively impact the seed purity of cultivars. It is thus imperative to increase our understanding of how pollinators impact gene flow from GE crops.

A standard method of limiting gene flow to ensure cultivar purity and limit adventitious presence is via isolation distance [21]. It is becoming more evident, however, that the optimal isolation distance may vary with pollinator species, because gene flow can vary with bee species [22, 23]. In alfalfa, for example, genes have been recovered farther distances in fields pollinated with honey bees, relative to fields pollinated with leafcutting bees [22]. These studies were done in different areas, and these findings have led to an isolation distance of 274m for leafcutting bees, and 4.8 km for honey bees [23]. Such differences among bee species implies that, in agricultural systems, one may be able to attribute different levels of gene flow risk (risk of GE gene escape) to distinct bee species. It thus becomes important to understand how such differences in gene flow among bee species could be explained by their respective foraging behaviors.

To examine this question, we studied the gene dispersal pattern of three bee species, the European honey bee, *Apis mellifera L*., the alfalfa leafcutting bee, *Megachile rotundata F*., and the common eastern bumble bee, *Bombus impatiens* Cresson foraging on *Medicago sativa* L. flowers. To assess the dispersal of genes by the three bee species, we used *Medicago sativa* L. plants carrying three copies of the glyphosate resistance (GR) allele as pollen donors, and conventional (non-GR) *M*. *sativa* plants as pollen recipients. We describe the relationship between the probability of getting a GR seed in a pod, and the order a flower is visited in succession by a bee or the cumulative distance it travels over a foraging bout. We refer to this relationship as a “seed curve” and we quantify the “seed curve” for each bee species. In addition, to further understand the pattern of GR seeds set by these three bee species, we contrasted different foraging metrics of the bee species, such as the number of flowers visited and tripped, and the number of GR seeds per foraging bout. Finally, we examined how the gene flow predictions based on the seed curves compared to gene flow estimates previously obtained in seed-production fields for these bee species. Understanding how seed curves vary among pollinator species can increase our understanding of why distinct pollinators have different impact on gene flow, and help determine whether some pollinator characteristics are linked to a greater ability to disperse genes. Such knowledge would help biotechnology regulators as they refine the rules for isolation distances between fields pollinated by specific pollinators. The rapid development of novel genome editing technologies and the eminent release of new GE varieties has heightened the importance of understanding how distinct bee species differentially affect gene flow, coexistence, and the spread of GE genes to sexually compatible feral and wild populations.

## Materials and methods

### Study system

*Medicago sativa* L. (Fabaceae), known as alfalfa or lucerne, is a tetraploid, self-compatible, perennial legume, widely cultivated worldwide. Flowers are organized into clusters or racemes and range in color from pale to dark purple [10]. Alfalfa relies mainly on bees for pollination and subsequent seed set, and a tripping mechanism enables pollen transfer. When a pollinator presses the two lower petals that form the keel, the enclosed stamens and pistil are forcefully released, and pollen is deposited on the pollinator’s body in a process called tripping. At the same time, pollen already on the pollinator’s body gets deposited on the stigma. Flowers that are not tripped do not set or sire seeds.

While alfalfa does not have sexually compatible wild relatives in the United States, feral alfalfa populations are relatively common near alfalfa seed production areas [16]. Moreover, the GR gene has been detected in a significant number of these feral populations and the gene can persist over time [16, 24]. The GR gene has also been identified in feral alfalfa populations in areas where only hay is produced [Brunet (unpublished data)]. Therefore, the GR gene can move not only among cultivated fields but also to feral populations and it does persist in the wild.

Managed pollinators in alfalfa seed production in the United States include the alfalfa leafcutting bee, *M*. *rotundata* and the European honey bee, *A*. *mellifera*. The strict habitat requirement of the alkali bee, *Nomia melanderi* Cockerell, restricts its use to the Walla Walla valley in Washington [23]. Wild bees, including the common eastern bumble bee, *B*. *impatiens*, also visit alfalfa flowers [25]. Wild bees have been shown to contribute to alfalfa pollination in Argentina [26] although this has not been reported for the United States. While bumble bees are used as pollinators in alfalfa breeding programs, they are not used in alfalfa seed production in the United States. These distinct bee species trip flowers at different frequencies (*i*.*e*. tripping rates, or the proportion of visited flowers that are tripped by a pollinator) [6, 27], and a higher tripping rate is linked to a greater seed set per bee visit [10]. While alfalfa leafcutting bees have high tripping rates and are efficient pollinators, honey bees learn to avoid the tripping mechanism and often become nectar robbers by drawing nectar from the side of the flower.

### Experimental design

**Fig 1:**
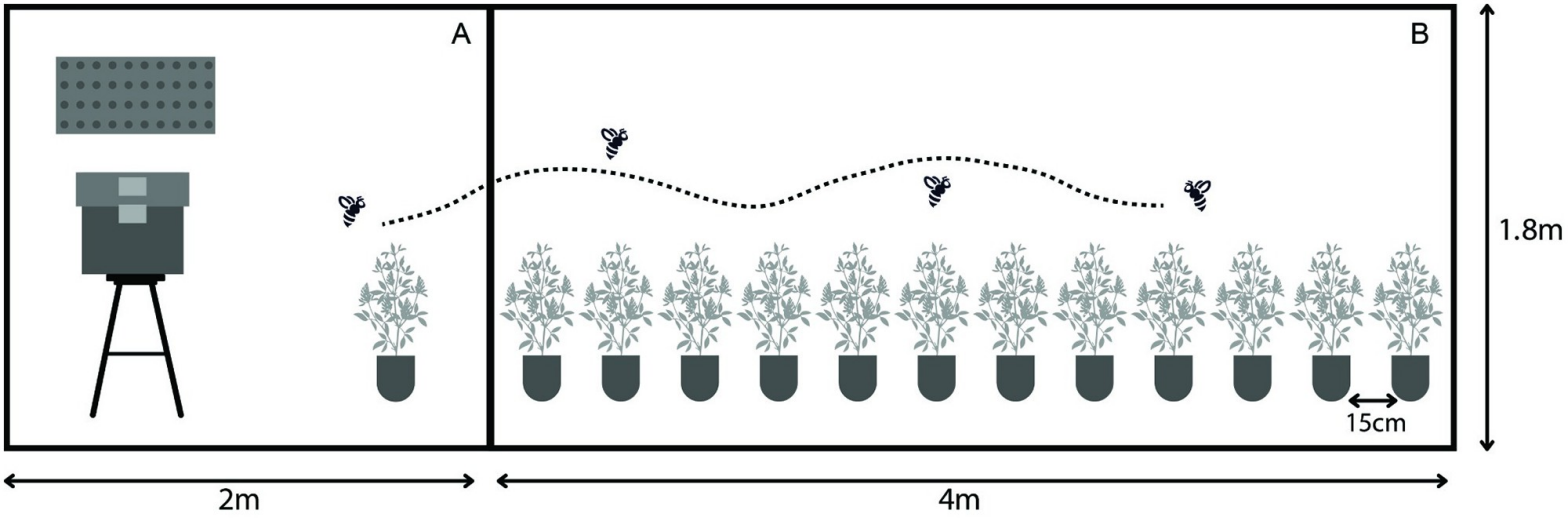
Experimental set up. A bee hive, or nesting board with bees, was kept in a small cage (A) adjacent to a large cage (B). Prior to trials, bees were trained to forage on conventional alfalfa plants in the small cage. During a trial, glyphosate-resistant (GR) donor plants were presented to a bee inside the small cage. After a bee tripped five flowers on the donor plants, it was moved to the large cage to forage on the conventional plants.

The experiments were carried out inside cages set up in a greenhouse room at the University of Wisconsin Walnut Street Greenhouse in Madison, WI, USA. A smaller 2.0 x 2.0 x 1.8 m cage adjoined a larger 4.0 x 2.0 x 1.8 m cage (Fig 1). The frames of the cages were made of PVC tubing and were covered with mosquito netting (Skeeta, Inc., Bradenton, FL, USA) which was also used to separate the two adjacent cages. Glyphosate-resistant (GR) alfalfa plants, also known as Roundup Ready (RR) alfalfa, were placed in the small cage and used as pollen donors in the experiment. Conventional alfalfa plants were placed in the large cage and used as pollen recipients.

The GR plants used as pollen donors had a single copy of the GR gene, and at least three copies of the GR allele. Because alfalfa is a tetraploid, all pollen grains of plants with at least three copies of the GR allele will carry at least one GR allele. Because the GR allele is dominant, one copy of the gene suffices for the expression of glyphosate resistance in the seeds and seedlings. While the GR gene is hemizygous when inserted, crosses are made between plants to increase the number of copies of the GR allele in plants that carried only one inserted copy of the GR gene. The number of gene and allele copies is verified by the proportion of GR seeds produced in crosses and via quantitative PCR.

Trials were run for one bee species at a time. Prior to starting a set of trials, bees were trained to visit alfalfa plants in the small cage, using several blooming conventional alfalfa plants. For bumble bees, we used a hive containing 75–100 workers (Koppert Biological Systems, Howell, MI, USA) and for honey bees, a four-frame nucleus honey bee colony (Gentle Breeze Honey, Inc., Mount Horeb, WI, USA). For leafcutting bees, 30–50 bees, incubated from commercially available cocoons, were released into the training cage every two days for the duration of the experiment. Both male and female leafcutting bees were released in the small cage to allow mating, because mated females collect pollen and nectar to provision their eggs. Only female bees were used in the trials. A 30 x 50 cm nesting board was placed in the small cage to permit leafcutting bees to nest. The hive or nesting board was set on the side of the small cage opposite the opening to the large cage (Fig 1).

Trials began after bees foraged consistently on alfalfa in the training cage. Prior to starting a trial, alfalfa plants used for training were removed from the small cage and bees were not allowed to visit any plant for at least 15 hours. We could close the bumble bee hive the evening prior to a trial, but honey bees and leafcutting bees remained free inside the cage. However, in the absence of plants, most honey bees would stay inside the hive and leafcutting bees would lay still on the netting. To set up a trial, two flowering GR alfalfa plants, used as pollen donors, were placed in the small cage. In addition, 12 conventional flowering alfalfa plants, used as pollen recipients, were placed in a linear array in the larger cage, each plant separated by 15 cm (Fig 1). Floral display size on recipient plants was standardized to 5–6 racemes per plant and 4–6 untripped flowers per raceme. A thin thread of a different color was used to distinguish the five racemes on each recipient plant, and each flower within a raceme was marked with a small dot of a distinct colored paint. Based on our observations, these markings did not affect bee behavior.

### Bee observations

Only one individual bee was tested in the large cage at any given time. For bumble bee, one female bee was released from the colony into the small cage. After the bumble bee tripped five GR flowers from the donor plants in the small cage, the screen separating the cages was lifted and the bee was moved to the larger cage. For honey bees and leafcutting bees, the first female bee to start foraging on the donor plants in the cage was tracked until it tripped five flowers. The bee was then caught in a centrifuge tube and moved to the large cage. Tripping five flowers required visiting, on average, between six and 14 flowers, depending on the bee species.

As soon as a bee started foraging on the conventional plants in the large cage, we used a voice recorder to note each plant, raceme, and flower visited in succession by the bee, for the duration of the foraging bout. A foraging bout could start from any plant position and revisits to plants and flowers were permitted. For the social bees, a foraging bout ended when the bee started flying back towards the hive, and for the leafcutting bees a bout ended when the bee landed for more than two minutes without grooming. Prior experience indicated that leafcutting bees were unlikely to keep foraging after this two-minute rest period.

### Flower order, distance traveled between successive flowers, and fruit collection

Each flower visited in succession during a foraging bout was given a flower order, corresponding to the order in which the flower was visited by the bee. At the end of each trial, we measured the distance between all pairs of flowers visited in succession by the bee. Each tripped flower was marked with a numbered tag and periodically checked for pod (fruit) development. Untripped flowers do not form pods because they remain closed, with no pollen being removed from the anthers or deposited on the stigmas. Pods were collected 4–6 weeks following a trial, when seeds were mature. Each individual pod with the associated tag was kept in a separate coin paper envelope and transferred to a refrigerator until pods could be hand-threshed to extract the seeds, and seeds could be counted and tested for the presence of the glyphosate resistance gene.

### Detection of the glyphosate resistance gene

Seeds collected from the bumble bee and leafcutting bee trials were tested for the presence of the GR gene using a seedling phenotypic assay, combined with test strips [28]. The phenotypic assay is a modification of a protocol originally developed by [29] to distinguish between conventional and GR seedlings. The test strips, lateral flow immunoassay AgraStrip^®^ RUR Seed and Leaf TraitChek™ (Romer Labs Inc., Union, MO, USA), detect the CP4 EPSP protein. Because the incubator needed to grow the seedlings was not accessible during the COVID-19 stay at home period, seeds collected from the honey bee trials were all individually tested using the test strips.

For the phenotypic assay, seeds were placed in petri dishes lined with filter paper and watered with a low-concentration (80 ppm) glyphosate solution. The petri dishes were placed in an incubator set at 20°C, under a 16h light: 8h dark regime for 14 days. Three days following the initial set-up, seedlings were watered again with the low-concentration glyphosate solution. Thereafter, seedlings were watered with a 0.1% Plant Preservative Mixture (PPM, Caisson Laboratories, Inc., Smithfield, UT) deionized water solution to prevent fungal growth. At the end of the 14-day period, seedlings were evaluated for traits associated with glyphosate resistance, such as increased root length, smaller root width, and development of secondary roots or root hair [28, 29]. When a seedling was categorized as positive using the phenotypic assay, the presence of the GR gene in the seedling was confirmed using a test strip [16]. Seedlings scored as conventional based on the phenotypic assay were bulk-tested in groups of twenty with the test strip. When a positive result was detected in a bulked sample, each seedling was individually tested with a test strip using its remaining cotyledons.

### Model selection

We used Generalized linear mixed models (GLMM) with a binomial error distribution and a logit link function to examine the relationship between the logit of the probability of getting GR seeds in a pod (log [p/(1-p)]) with increasing order of flower visited or cumulative distance traveled by a bee in a foraging bout. The binomial error distribution reflected the data structure which included binomially distributed constrained counts (presence or absence of a GR seed). The logit transformation limited the values of p between 0 and 1. Here, p is the probability of getting a GR seed in a pod or the modelled proportion of GR seeds in a pod and (1-p) is the probability of getting a conventional (non-GR) seed in a pod. The origin for distance was the first flower visited and we added incrementally the distances between pairs of flowers visited in succession during a foraging bout.

The first model fitted to the data assumed a linear relationship between the logit of the dependent variable and each predictor (independent) variable (order of flower visited or cumulative distance traveled in a foraging bout). Because the logit of the probability of getting GR seeds in a pod may exhibit a different rate of decline as a function of the independent variable, we evaluated two additional models. The second model used the natural log of flower order or distance traveled, and the third model utilized the square root of flower order or distance traveled. In these two models, the relationship between the logit of the dependent variable and the independent variable is no longer linear. These models are abbreviated in the remainder of the manuscript, as the (1) “standard”, (2) “log”, and (3) “sqrt” models. Each model was fitted to the combined runs for each bee species using the “glmer” function in the lme4 package [30] in R [31].

To take into account the variation among runs (foraging bouts), for each of the three types of models just described–standard, log or sqrt–we fitted a mixed effect model with either random intercept or both random intercept and random slope. Thus, for each bee species and each predictor variable (flower order or cumulative distance), we fitted six different models. Each model has a fixed (population means) intercept and slope. In addition, the random intercept corresponds to a random run effect and represents variation among individual foraging bouts in the logit of the probability of getting GR seeds set in the first flower visited. The random slope considers variation among runs in the linear decline in the logit of the probability of getting GR seeds in a pod with increasing flowers visited or distances traveled. Because individual bees were not marked in this study, we are examining variation among foraging bouts, but in a study with marked bees and a single foraging bout per bee these variables would describe the bee-to-bee variation.

The six models per predictor variable per bee species were compared using the Akaike Information Criterion (AIC). The model with the lowest AIC was considered the best fit to the data and models were considered distinct if the difference in AIC between models was greater than two [32]. We examined a total of 36 models, two predictor variables times three bee species times the six models described above. The figures, presented for the best models, illustrate the back transformed logit, *i*. *e*. p or the probability of getting GR seeds in a pod. Logits were back transformed using the following equation: p = exp (a + b predictor variable) / [1 + exp (a + b predictor variable)]. Individual data points on the figures represent the observed proportion of GR seeds in a pod, for each pod within each of the foraging bouts, where a pod is a fruit developed following bee visits to a flower.

Analyses were performed on tripped flowers because untripped flowers do not set seeds. The order of visit of tripped flowers was determined and cumulative distance calculated between tripped flowers visited in succession. The cumulative distance was converted from centimetres to metres to facilitate model fitting. Because a flower is only tripped once, if a flower was visited more than once, we assumed it got tripped during the first bee visit. A previous study indicated no increase in the number of pollen grains deposited over repeated visits [33].

We obtained the best model to explain the data (proportion of GR seeds in a pod) for each bee species separately. We can only compare the model parameters between bee species when the same type of model best explains the data for these bee species. When these conditions are met, models can be compared between bee species by adding bee species and the interaction between bee species and either flower or distance traveled as factors in the model. This approach fits the same model type to each bee species and compares their slopes and intercepts. A statistically significant species effect indicates distinct intercepts between bee species, while a statistically significant interaction illustrates different slopes. However, when different model types best fit the data for each bee species, this statistical model cannot be used to compare parameters between bee species and the intercepts and slopes are contrasted visually.

### Comparing foraging metrics among bee species

We compared different foraging metrics among the three bee species using one-way analysis of variance (ANOVA), followed by multiple pairwise-comparisons with Tukey HSD tests in R [31]. These foraging metrics included the total number of flowers visited per foraging bout, the proportion of flowers visited in a foraging bout that were tripped, foraging bout duration, and the cumulative distance traveled per foraging bout. A foraging bout starts with the first flower a bee visits in the array, and ends when the bee leaves the array. We also determined the proportion of flowers tripped in a foraging bout that set seeds, and the total number of seeds set and of GR seeds set per pod, and per foraging bout.

## Results

### Bumble bee models

**Fig 2:**
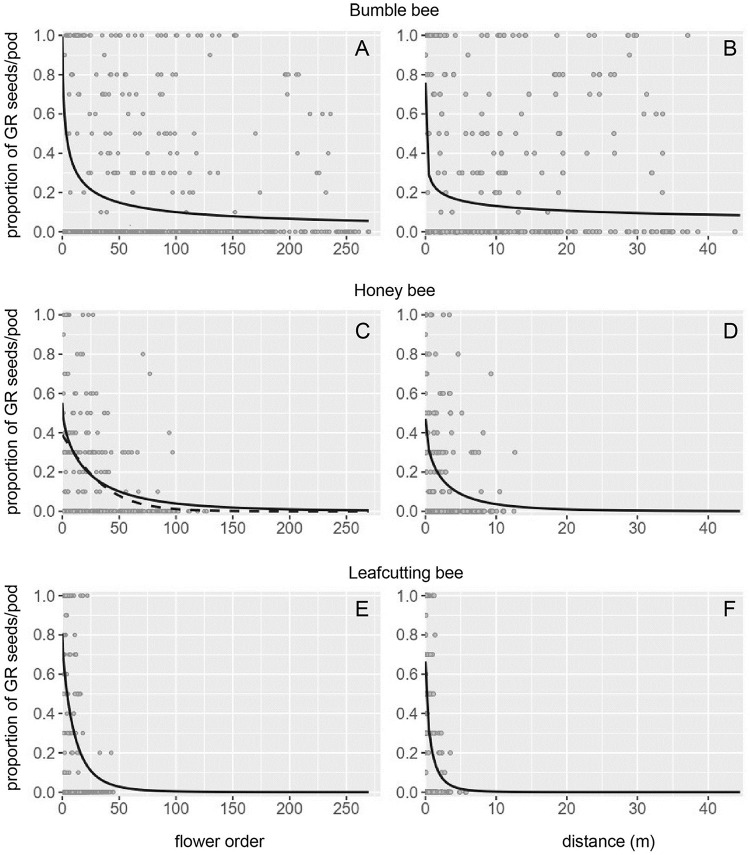
Probability of getting GR seeds in a pod with increasing flower order or distance traveled (m) by a bee. Probabilities represent back transformed logits (see text for details). For bumble bee, the log model with random intercept was the best model for both (A) flower order and (B) cumulative distance; for honey bee, the square root (solid line) and the standard (dashed line) models with random intercept were equally good candidates for (C) flower order while the square root model with random intercept was the best model for (D) distance; for leafcutting bee, the square root model with random slope and random intercept was the best model for both (E) flower order and (F) distance.

We had 12 foraging bouts (runs) and 322 observations (pods with seeds). The logistic regression analysis only considers the pods that set seeds. For both flower order and cumulative distance, the log-odds ratio or logit of getting GR seeds in a pod was best described by the log model with random intercept, log_e_ (p/(1-p) = intercept—slope log_e_ flower order (or distance) (Table 1). The intercept and slope with standard errors for the fixed effects of the best model for flower and for distance are presented in Table 2. These standard errors (SE) illustrate the accuracy of the parameters. For the flower model, the standard deviation (SD) associated with the random effect for the intercept was 1.41 and illustrates variation among foraging bouts in the logit of getting GR seeds in the first flower visited in the foraging bout. For distance, the standard deviation for the random intercept was 1.50. The probability of getting GR seeds in a pod decreased with successive flowers visited for flower order (Fig 2A), and a similar trend was observed for the cumulative distance traveled by a bee (Fig 2B).

**Table 1:** Akaike information criterion (AIC) values for flower and distance models for three bee species.

Bee species	bumble bee	honey bee	leafcutting bee
	(N = 322)	(N = 268)	(N = 202)
**Flower Models**			
Standard random intercept	853.2	**581.2**	519.7
Standard random slope and intercept	840.8	584.6	511.5
Log random intercept	**818.0**	590.7	524.1
Log random slope and intercept	821.8	594.2	513.9
Square root random intercept	833.8	**579.6**	517.7
Square root random slope and intercept	828.9	583.1	**508.1**
**Distance Models**			
Standard random intercept	861.1	585.8	523.3
Standard random slope and intercept	840.2	587.0	504.7
Log random intercept	**821.3**	613.7	542.2
Log random slope and intercept	823.8	616.5	537.3
Square root random intercept	839.9	**581.8**	513.4
Square root random slope and intercept	826.0	585.8	**498.7**

The models describe the logit of the probability of getting glyphosate-resistant (GR) seeds in a pod with increasing order of flower visited or cumulative distance traveled in a foraging bout for three bee species. A foraging bout starts with the first flower a bee visits and ends when the bee leaves the array. The models with the lowest AICs are in bold. The sample size N represents the number of observations or the number of pods (fruits) with seeds over all foraging bouts for each bee species.

**Table 2:** Fixed effect estimates for the best flower and distance model(s) for bumble bees (BB), honey bees (HB), or leafcutting bees (LCB).

Bee Species	Best Model	intcp	SE	slope	SE
	**Flower models**				
BB	Log RI	0.80	0.53	-0.65	0.09
HB	Standard RI	-0.45	0.25	-0.04	0.00
HB	Square root RI	0.22	0.28	-0.34	0.04
LCB	Square root RIRS	1.45	0.56	-0.71	0.23
	**Distance models**				
BB	Log RI	-1.13	0.47	-0.33	0.05
HB	Square root RI	-0.08	0.28	-1.01	0.12
LCB	Square root RIRS	0.75	0.38	-2.16	0.54

The models describe the log odds ratio (logit) of the probability of getting glyphosate-resistant seeds in a pod with increasing order of flower visited or cumulative distance traveled. The intercept (intcp) and the slope (fixed effects), with associated standard errors (SE) for the best models. The variable RI stands for random intercept and RIRS for random intercept and random slope.

### Honey bee models

The data for honey bees included 14 foraging bouts and 268 observations. We obtained two best models for flower order, the square root model with random intercept, log_e_ (p/(1-p)) = intercept—slope sqrt flower order, and the standard model with random intercept, log_e_ (p/(1-p)) = intercept—slope flower order (ΔAIC < 2) (Table 1). The intercept and slope with standard error for the fixed effects of the best model are presented in Table 2 The standard deviation associated with the random intercept was 0.75. For distance, the square root model with random intercept was the best model (Table 1), with parameters for the fixed effects of the model presented in Table 2. The standard deviation associated with the random intercept was 0.82. In all cases, the probability of getting GR seeds in a pod decreased as more flowers were visited in succession by a bee (Fig 2C), or longer cumulative distances were traveled (Fig 2D).

### Leafcutting bee models

We obtained 27 foraging bouts and 202 observations for leafcutting bees. The square root model with random slope and random intercept best explained the logit of the probability of getting GR seeds in a pod, for both successive flowers and for cumulative distance (Table 1). The parameters for the fixed effects of the model (intercept and slope) with respective standard errors are presented in Table 2. For flower order, the standard deviations were 2.12 for the random intercept, and 0.89 for the random slope. For distance, the standard deviation associated with the random intercept was 1.51 and with the random slope 1.90. The probability of getting GR seeds in a pod decreased with successive flowers visited (Fig 2E), and with longer cumulative distance traveled by a bee (Fig 2F). For leafcutting bees, in addition to the random intercept, the random slope variable, *i*. *e*. considering among foraging bout variation in slope, improved the fit of the model to the data.

### Comparing models among bee species

Because the type of the best model (standard, log or sqrt), for either flower order or distance, differed among bee species (Table 2), we could not apply the same model type to statistically compare the intercepts and slopes among bee species. Therefore, to address potential differences among bee species, we visually contrasted the graphs illustrating the probability of finding a GR seed in a pod with successive flowers visited and cumulative distance traveled (Fig 2). Bumble bees had a fairly steep rate of decay in the probability of getting a GR seed in a pod (slope), but the probability levelled off after visiting approximately 30 flowers or traveling five meters, creating a distribution with a long tail (Fig 2A and 2B). When bumble bees moved pollen from flower to flower, GR seeds could still be found with a low probability in a pod after a bee visited 250 flowers or traveled over 40 meters. Honey bees were intermediate between bumble bees and leafcutting bees, with a negligible probability of getting a GR seed in a pod after a honey bee visited approximately 100 flowers or traveled around 15 meters (Fig 2C and 2D). The rate of change in the probability of getting a GR seed in a pod with increasing flower order or distance was steep when pollen was moved by leafcutting bees (Fig 2E and 2F). The probability of getting a GR seed in a pod was negligible after a leafcutting bee visited 50 + flowers or traveled five + meters (Fig 2E and 2F).

### Foraging metrics

All foraging metrics, except the proportion of tripped flowers that set seeds, varied among bee species (Table 3). Bumblebees visited more flowers, spent more time foraging, and traveled greater cumulative distance in a foraging bout relative to honey bees or leafcutting bees (Table 3). Leafcutting bees tripped the most flowers and set a greater proportion of GR seeds per foraging bout relative to honey bees and bumble bees (Table 3). Honey bees set the most mature seeds per pod and were otherwise similar to either bumble bees or leafcutting bees, depending on the variable examined (Table 3). Although leafcutting bees set a greater proportion of GR seeds per foraging bout, they set fewer seeds, and the lowest number of GR seeds per foraging bout (Mean ± SE) (9.22 ± 1.31), relative to bumble bees (31.08 ± 11.87), and honey bees (17.07 ± 3.49). However, per pod, leafcutting bees set slightly more GR seeds (1.47 ± 0.14) relative to honey bees (1.03 ± 0.20) and bumble bees (0.98 ± 0.31).

**Table 3:** Differences in foraging metrics (per foraging bout) among bee species with respective means and standard errors.

Foraging metrics	Overall model		Bumble bee		Honey bee		Leafcutting bee	
	F	Pr > F	Mean	SE	Mean	SE	Mean	SE
Number of flowers visited	16.70	< 0.0001	99.8^a^	23.56	57.4^b^	8.96	16.4^b^	1.79
Proportion of visited flowers that were tripped	8.83	0.0005	0.72^b^	0.04	0.66^b^	0.06	0.87^a^	0.02
Foraging bout duration (min)	7.43	0.0015	17.7^a^	3.20	9.5^b^	1.51	8.6^b^	1.04
Cumulative distance (cm)	23.07	< 0.0001	1697^a^	387.60	478^b^	109.89	140^b^	26.91
Proportion of tripped flowers setting seeds	1.53	0.226	0.58^a^	0.08	0.73^a^	0.05	0.69^a^	0.04
Mature seeds per foraging bout	5.99	0.0046	105.3^a^	37.01	93.6^a^	23.51	28.5^b^	5.56
Mature seeds per pod	3.32	0.0441	3.3^b^	0.31	4.5^a^	0.29	3.5^b^	0.27
Proportion of GR seeds per foraging bout	5.42	0.0074	0.25^b^	0.06	0.25^b^	0.06	0.48^a^	0.05
Proportion of GR seeds per pod	4.18	0.021	0.24 ^ab^	0.07	0.21^b^	0.05	0.43^a^	0.05

Degrees of freedom for the model were df = 2 for the numerator and 50 for the denominator for all foraging metrics. The overall model indicates whether bee species statistically affected the foraging metric. Different letters indicate statistically significant differences in the foraging metrics among bee species based on the result of pairwise-comparisons with Tukey HSD tests. A foraging bout starts with the first flower visited by a bee and ends when the bee leaves the plant array. The abbreviation GR stands for glyphosate-resistant.

## Discussion

Leafcutting bees have the steepest rate of decay in the probability of setting a glyphosate-resistant seed, generate the lowest number of GR seeds per foraging bout (13.7), and carry these GR genes via pollen the shortest distances. Honey bees and bumble bees set similar number of seeds, and similar number of GR seeds per foraging bout (bumble bees: 26.3 GR seeds; honey bees: 23.4). Bumble bees have the dispersal curve with the longest tail, and moved more GR genes via pollen the longest distances. Honey bees moved genes via pollen intermediate distances. If we assign a probability of moving genes to each bee species, leafcutting bees would have the lowest probability, and bumble bees would have the highest.

While the current study examined the decrease in the probability of getting a GR seed in a pod over an average foraging bout, the findings are consistent with pollinator-mediated gene flow estimates obtained in alfalfa seed-production fields [22, 34–36]. The gene flow estimates obtained from seed production fields indicated lower distances traveled by GR genes when alfalfa leafcutting bees, relative to honey bees, were used as managed pollinators. In the USA, bumble bees are not used as managed pollinators in alfalfa seed-production fields, are not important pollinators in these fields, and we are not aware of gene flow estimates for bumble bees in seed production fields. For leafcutting bees and honey bees, predictions based on seed curves are consistent with gene flow estimates obtained in seed production fields [22]. This association is important because it supports a relationship between bee behavior within foraging bouts, and gene flow estimates at the field level. Such a connection is reminiscent of the relationships previously observed between behavior of birds observed on a small scale and seed dispersal at the landscape level [37, 38]. These latter studies simulated the small-scale movements of birds, based on perching time, move length, and move direction, to predict bird-mediated seed dispersal of a plant species. Moreover, these results suggest how differences in seed curves among bee species can inform on the gene flow potential of these bee species. The observed differences in gene flow among bee species are further supported by the results of a 1965 experimental study that compared gene flow distances using a white flowered alfalfa morph as pollen recipient, and variegated variety as pollen donor [39]. In this latter study, some bumble bee species were used and found to set seeds the farthest distances, followed by honey bees, and least leafcutting bees. Thus, gene flow predictions based on seed curves fit the observed gene flow data, linking bee behavior within foraging bouts to gene flow estimates at the field level.

Patterns of seed dispersal are linked to patterns of pollen dispersal, although the two will differ because not all pollen grains deposited on a stigma will set viable seeds. Patterns of pollen dispersal can be influenced by the frequency and location of pollen accumulation on the body of the pollinator, and by the grooming behavior of the pollinator [40]. A previous study in alfalfa determined that bumble bees moved pollen further than leafcutting bees and deposited more pollen grains on stigmas over a foraging bout. These differences could not be explained by distinct grooming frequency between the two bee species, but were correlated with their tripping rates [33]. When using GE plants as pollen donors and conventional plants as pollen recipients, one expects pollen of the GE donors to get depleted from the bee’s body as a bee visits and trips more consecutive flowers, while pollen from the conventional plants would accumulate. Grooming could lead to a low probability of resurfacing of the GE pollen [41]. A bee species with a higher tripping rate should deplete its GE pollen faster than a bee species with a lower tripping rate and the pollen should move shorter distances because pollen only gets deposited on tripped flowers [6]. The lower the tripping rate, the greater the number of untripped flowers visited, which increases the distance traveled by the GE pollen (on the body of the bee) before it gets deposited on the stigmas. For plant species without a tripping mechanism, a bee with a high tripping rate would be equivalent to an efficient pollinator. We thus expect efficient pollinators to move genes shorter distances. The tripping rate varies among the three bee species, with leafcutting bees typically tripping a greater proportion of visited alfalfa flowers relative to honey bees or bumble bees [6, 25]. Honey bees learn to avoid the tripping mechanism through experience, stealing nectar from the side, and naïve bees tend to trip more flowers than experienced bees [42]. However, the tripping rate of honey bees was quite high in the current study, likely because no other sources of pollen were made available and the bees had to gather their pollen from *M*. *sativa* flowers. In fact, in the current study the tripping rate of honey bees was similar to the tripping rate of bumble bees, which itself was higher than under field conditions (honey bee in the field: 22.7% and bumble bee: 50.7% over two years [6]. While the high tripping rate of leafcutting bees could help explain its steep decline in the probability of setting a GR seed in a pod, tripping rate alone was not sufficient to explain the long dispersal tail of bumble bees. The tripping rate is constant for a bee species, and therefore tripping rate alone cannot explain why the proportion of GR seeds in a pod following bumble bee visits did not keep declining as it did for the other two bee species, resulting in a long dispersal tail.

Other physical and behavioral differences among bee species help explain observed differences in seed curves. Previous studies have linked bee body sizes with their foraging ranges, defined as the maximum distance a bee species would travel from its nest or hive, based on homing, feeders training, or bee dance interpretation [43–45]. Larger bees fly longer distances although honey bees with their waggle dance have been predicted to forage farther distances relative to bumble bees despite their smaller size [46–48]. However, bee foraging range is quite different from the distance traveled while a bee is foraging for resources, and the latter measure is most likely to affect how pollen and the resulting seeds are spread [49]. In the current study, bumble bees, the largest of the three bees, visited the most flowers per foraging bout, and traveled the longest total cumulative distance. The smallest bees, the leafcutting bees, visited the least flowers, and traveled the shortest total cumulative distance. Honey bees were intermediate for both flowers visited and total cumulative distance traveled. The ranking of their body sizes correlates with the ranking of the distances traveled during a foraging bout. In addition, a field study recorded bumble bees traveling the longest net distances in a foraging bout as they foraged in alfalfa patches, followed by honey bees, while leafcutting bees traveled the shortest distances [6]. A net distance represents the distance between where a bee started and ended a foraging bout; it represents the straight-line distance between the first and last visited racemes in a foraging bout. Future studies should increase the sample size of bee species to further strengthen the correlation. Available data indicate how bee body size is linked not only to foraging range, but also to the distance bees traveled in a foraging bout, the latter measure being more relevant to pollen dispersal and gene flow.

Bee body size may also affect the size of the pollen load carried by the bee, which in turn can influence gene flow. Using radio frequency identification (RFID), Minahan and Brunet [50] determined that, despite spending similar amount of time during a foraging trip, bumble bees brought heavier pollen sacs back to the hive relative to honey bees. A foraging trip included the time between leaving and returning to the hive. Although pollen present in pollen baskets is not available for pollination, the heavier loads carried by bumble bees may also reflect a greater pollen load on the parts of their body that come into contact with plant stigmas. Bee species vary in the size of their pollen loads for specific plant species [51], and, in this respect, bumble bees deposited more GE pollen grains during an average foraging bout relative to leafcutting bees [33], and had more GE pollen grains left on their body at the end of a foraging bout [Brunet (unpublished data)], suggesting greater pollen loads on their bodies. In addition, in the current study, bumble bees set the most GE seeds in a foraging bout, followed by honey bees, and least leafcutting bees, supporting a correlation between bee body size and the total number of GE seeds set in an average foraging bout. Bee body size is associated not only with bee movement [6], but also with pollen dispersal [33], and the resulting seeds (this study). It is also worth noting how a heavier pollen load on the bee’s body, combined with a larger number of flowers visited per foraging bout, and longer distances traveled, help explain the longer tail observed in the seed curve when bumble bees moved GE pollen, relative to the other two bee species.

Distinct pollinators have been previously shown to differentially affect plant reproductive success, pollen dispersal, and selection on floral traits [9, 10, 33, 52–55]. Here, we show how distinct bee species have different seed curves, which predict the relative gene flow of these bee species in the field. In agriculture, a low probability of moving genes is associated with a low gene flow risk [6]. Bees with lower gene flow risk such as leafcutting bees would generate the least adventitious presence, and minimize introgression and spread of GE genes to wild or feral cross-compatible populations [11]. In natural populations, pollinators with a high probability of moving genes such as bumble bees would be linked to a greater impact on homogenising the genetic diversity of natural plant populations, and thus limiting genetic differentiation [1]. Distinct bee species will have differential impact on the spread of GE genes in agriculture, and on the genetic differentiation of wild populations.

## Conclusions

The major goal of this study was to describe and compare the seed curves of three bee species foraging on *M*. *sativa* flowers. We then examined whether the differences in seed curves could explain differences in gene flow estimates obtained in the field; and identified bee characteristics that could help explain the differences in seed curves. Based on their seed curves and total number of GR seeds produced within foraging bouts, leafcutting bees have the lowest probability of moving genes, and move genes the shortest distances; honey bees are intermediate; and bumble bees move genes the farthest and set the most GR seeds per foraging bout. Shorter isolation distances are used for leafcutting bees in alfalfa seed production fields, and this study helps explain why leafcutting bees move genes via pollen shorter distances. The differential seed curves of bee species, reflecting within foraging bout patterns, translate into gene flow estimates at the landscape level, linking bee behavior at a small scale to movement at the landscape level. Moreover, characteristics of the bees such as tripping rate and body size helped explain differences among seed curves and gene flow potential. Future studies should keep examining which bee characteristics best predict their potential for gene flow. Such information benefits farmers and regulators aiming at reducing adventitious presence in agriculture, and increases our understanding of how bee species influence the genetic structure of plant populations.
